# A new approach to comprehensively evaluate the morphological properties of the human femoral head: example of application to osteoarthritic joint

**DOI:** 10.1038/s41598-020-62614-7

**Published:** 2020-03-26

**Authors:** M. Ryan, L. Barnett, J. Rochester, J. M. Wilkinson, E. Dall’Ara

**Affiliations:** 1 Department of Oncology and Metabolism, Mellanby Centre for bone Research, University of Sheffield, Sheffield, UK; 2 INSIGNEO Institute for in silico Medicine, University of Sheffield, Sheffield, UK; 3 Academic Unit of Medical Education, Medical School, University of Sheffield, Sheffield, UK

**Keywords:** Bone, Tissues

## Abstract

Osteoarthritis affects the morphological properties of the femoral head. The goal of this study was to develop a method to elucidate whether these changes are localised to discrete regions, or if the reported trends in microstructural changes may be identified throughout the subchondral bone of the human femoral head. Whole femoral heads extracted from osteoarthritic (n = 5) and healthy controls (n = 5) underwent microCT imaging 39 μm voxel size. The subchondral bone plate was virtually isolated to evaluate the plate thickness and plate porosity. The trabecular bone region was divided into 37 volumes of interest spatially distributed in the femoral head, and bone morphometric properties were determined in each region. The study showed how the developed approach can be used to study the heterogeneous properties of the human femoral head affected by a disease such as osteoarthritis. As example, in the superior femoral head osteoarthritic specimens exhibited a more heterogeneous micro-architecture, with trends towards thicker cortical bone plate, higher trabecular connectivity density, higher trabecular bone density and thicker structures, something that could only be observed with the newly developed approach. Bone cysts were mostly confined to the postero-lateral quadrants extending from the subchondral region into the mid trabecular region. Nevertheless, in order to generalise these findings, a larger sample size should be analysed in the future. This novel method allowed a comprehensive evaluation of the heterogeneous micro-architectural properties of the human femoral head, highlighting effects of OA in the superior subchondral cortical and trabecular bone. Further investigations on different stages of OA would be needed to identify early changes in the bone.

## Introduction

Measuring accurately the heterogeneous micro-architectural properties of human bones is important to evaluate bone strength^1^, the effect of musculoskeletal pathologies^2^ and of new interventions^3,4^. Recent improvements in micro computed tomography (microCT) imaging allows the scanning of large specimens at high resolution and the evaluation of morphometric and densitometric properties in different region of largely heterogeneous bones^5^. Nevertheless, analysis of the heterogeneous morphometric properties of complex structures within a large volume of interest remains challenging and in this manuscript an approach to comprehensively analyse the effect of osteoarthritis (OA) on a large portion of the femoral head will be presented.

In recent years, considerable emphasis has been placed on the role of subchondral bone in both the pathogenesis and initiation of the disease^6–8^. There is some evidence to suggest that distinct alterations in subchondral bone occur at different stages of the disease. Several studies have reported subchondral bone plate (SBP) thinning and loss of subchondral trabecular bone (STB) in early OA^9–12^, with some suggestions that subchondral bone changes may precede changes in cartilage^13,14^. However, the hallmark features of end-stage OA comprise a thickening of the SBP^12,15,16^, together with increased bone volume fraction, increased trabecular thickness, and decreased trabecular separation in the STB^17–20^, lower bone mineralisation^21,22^ and the presence of subchondral bone cysts (SBCs), and osteophytosis^23^. The aforementioned studies highlight overall trends in subchondral bone changes, but these studies have been primarily restricted to excised bone specimen cores, in most cases taken along the main loading axis of the bone. Bone adaptation theories suggest that the principal bone changes are likely to occur in the dominant loading zone (i.e. along the mean trabecular direction), however, it is possible that changes in gait and loading direction, in response to the pain associated with OA, may lead to altered distribution of stresses in the joint^24^. Identification of regional changes in bone density and micro-architecture, and how these relate to the primary loading surface may help identify the effect of OA on the bone properties, and therefore the pathophysiology of OA.

One of the first studies to look at regional differences in bone properties within the femoral head was performed by Li and Aspden^21^. The authors measured mechanical and material properties of excised bone cores within the femoral head, across 5 different regions, in OA, osteoporotic (OP) and healthy specimens; demonstrating spatial heterogeneity in bone density and stiffness, in the OA specimens. The sites were chosen specifically to represent regions subjected to different amounts of *in vivo* loading. Li, *et al*.^25^ explored subchondral bone changes with respect to the depth of bone relative to the loading surface, showing a distinct difference in micro architecture and bone remodelling between the deeper and more superior regions of subchondral bone. However, both studies were restricted to excised cores of trabecular bone at discrete locations.

A limited number of studies have performed spatial analyses on bone within the human femoral head^26^. Using peripheral quantitative computed tomography (pQCT), Tamaddon, *et al*.^22^ performed a mapping of volumetric bone mineral density (vBMD) over 36 regions, relative to the region of greatest cartilage damage; reporting a decrease in vBMD immediately adjacent to overlying cartilage defects, which continued deep into the bone, but an increase in vBMD in the bone surrounding SBP. While this approach showed heterogeneous vBMD across the femoral head, nothing is known about the heterogeneity of micro-architectural parameters in the cortical and trabecular bone, considered by some the main determinants of bone quality^27^. Chiba, *et al*.^28^ performed perhaps the most extensive evaluation of regional variations in trabecular bone microarchitecture, dividing the femoral head into 10 volumes of interest, looking at the influence of depth, as well as medio-lateral and antero-posterior influences. However, in their analyses, performed with high resolution peripheral pQCT (HRpQCT) with a spatial resolution of 88 μm, they did not measure in detail the properties of the cortical shell, probably due to the limited spatial resolution. Furthermore, the specimens analysed in that study were obtained from osteoporotic Japanese patients and no similar work has been extended to evaluate spatial differences in bone changes in OA. It is well accepted that subchondral bone changes play an important role in the pathogenesis of OA, so understanding the extent to which the diseased bone is essential to better understanding the underlying mechanisms of OA.

The aim of this study was therefore to develop a method that would allow a comprehensive evaluation of regional variations in bone morphometry within the femoral head. The implementation of such a systematic assessment would enable identification of any regions that are more disposed to different pathologies, and for this study it was applied as example to femoral heads from OA patients and healthy controls.

## Methods

### Materials

Five femoral heads were obtained from patients who underwent routine total hip replacement for clinically diagnosed OA at the Royal Hallamshire Hospital (Sheffield). A further five cadaveric specimens were obtained from five from subjects with no sign of bone and joint disease and were used as healthy controls (HC). All research procedures were approved by the ethic committee of the University of Sheffield (for HC; Reference Number: 014803) and by the South Yorkshire and North Derbyshire Musculoskeletal Biobank (for OA; Reference Number: STH15691). The research was performed in accordance with relevant guidelines and regulations of the university of Sheffield. Informed consent was obtained from all participants or their legal guardians. Due to the preliminary nature of the study, and subsequent small sample size, the two cohorts were unmatched; the demographics of the two groups were as follows: the OA donors had a mean age of 65.4 years (standard deviation (SD) = 9.7, range 52–82) comprising 4 females and 1 male; the HC cadaver donors had a mean age of 85.8 years (SD = 12.4, range 67–101) comprising 2 females and 3 males.

OA specimens were stored in 70% ethanol at the time of retrieval for at least one month in order to reduce the biological risk. HC specimens were treated with formalin and stored at −20C. On the day prior to scanning, OA specimens were removed from the ethanol solution, placed on absorbent paper for 30 minutes to remove excess liquid and potted in an acrylic resin (Technovit 4071, Kulzer GmbH, DE), then placed within a radio-transparent Perspex tube. The potting and placement in the radio-transparent tube were required for subsequent mechanical loading, which was performed as part of a separate study. Similarly, HC specimens were removed from the freezer on the day prior to testing and left in an airtight plastic container to defrost. On the day of scanning, specimens were fixed to the base plate of a custom developed imaging jig and placed in the scanning machine similarly to the OA specimens. The two types of storage were considered appropriate evaluate the morphometric properties of bone^29–31^.

### Imaging

MicroCT images were acquired (VivaCT 80, Scanco Medical AG, CH) with a voxel size of 39 × 39 × 39 μm^3^ and the following scanner settings: 70 kVp, 114 mA, 300 ms integration time. Scan lengths ranged from 39.23 mm (1006 slices) to 59.56 mm (1527 slices), taking between 67 and 95 minutes per scan. A third-order polynomial beam hardening correction, determined using a 1200 mg HA/cm3 wedge phantom^32^, was applied to all scans as recommended by the manufacturer.

### Image analysis

The method was developed by creating a spherical co-ordinate system with an origin based at the centre of the femoral head. The femoral head was then divided based on depth relative to the femoral head surface as well as by azimuth angle in the transverse plane and elevation angle in the sagittal and coronal planes. The details of this process are explained below.

**Figure 1:**
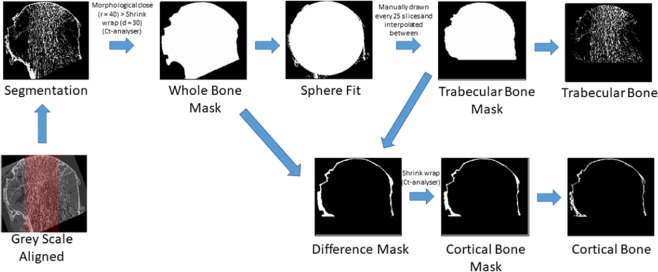
Flow diagram illustrating the methods to extract the trabecular bone cortical bone volumes for morphometric analysis. The mean trabecular direction (MTD) (highlighted in red) was aligned with the supero-inferior (SI) axis in the coronal and sagittal views. 16-bit grey scale images were binarised by implementing a global threshold based on the image stack histogram. A mask of the whole bone was used to determine the radius and centre of a best fit sphere within the femoral head. The centroid was used as the lower slice for the region over with the analysis was performed.

Reconstructed images were repositioned by aligning the mean trabecular direction (MTD) parallel to the supero-inferior (SI) axis in the central coronal and sagittal slices and the fovea capitis femoris was then aligned perpendicular to the antero-posterior (AP) axis on the coronal and sagittal views (Fig. 1)^28^. High frequency noise was removed by applying a Gaussian filter (support = 3 voxels, sigma = 1.2)^33^, followed by segmentation with a global threshold value (39,300 grey scale value, GSV). The threshold was chosen based on the histogram as the best value that allowed discrimination between bone matrix and marrow.

The binary images were used to create a “whole bone mask” of the entire bone volume by applying a closing algorithm (radius = 40 voxels) in 2D followed by region of interest (ROI) shrink wrap (stretch over holes = 30 pixels in 2D) in CTAn (v1.17.7.2, Bruker, US). To determine the volume to be analysed, a sphere was fit inside the whole bone mask volume using a custom Matlab script^34^ (R2017b, Mathworks, US), which provided the sphere centroid (x_c_, y_c_, z_c_) and radius (r_sphere_). The trabecular bone was delineated from the cortex by manually drawing a ROI from the first slice to be evaluated and every 25 axial sections (0.975 mm); the ROI was interpolated in between the manually drawn scans^35^ to create a “trabecular bone mask” (Fig. 1). For the OA specimens, the sphere fit routine was re-run once the trabecular bone mask was created, to ensure the volumes of interest were centered on the trabecular bone, and not affected by the presence of any osteophytes.

The volumes of interest (VOIs) were then created as follows. The cortical VOI for the healthy specimens was created by a Boolean subtraction between the “whole bone mask” and the “trabecular bone masks” (Fig. 1). For the OA specimens, the same operation was performed, which resulted in a mask containing both the cortical bone and osteophytes. The presence of osteophytes on the distal surface of the femoral heads made it difficult to delineate the outer border of the cortical bone, and impeded therefore the measurement of the cortical thickness. In order to accurately measure the cortical thickness, it was necessary to isolate just the cortical layer. Consequently, the cortical masks were generated for only the most proximal 257 slices (approximately 10 mm depth), which did not include any osteophytes.

The following micro-architectural parameters were measured in the cortical bone region: plate thickness (Pl.Th, μm), calculated using the local sphere-fitting method^36^, and total plate porosity (Pl.Po, %), calculated as the percentage of voxels defined as non-mineralised tissue within the VOI after segmentation^37^.

**Figure 2:**
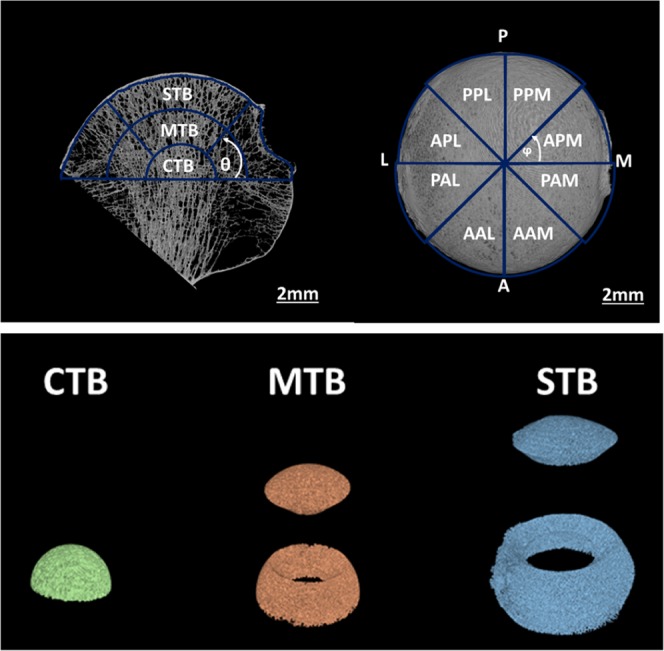
(Top): (left image): CTB = central trabecular bone, MTB = mid trabecular bone and STB = subchondral trabecular bone. The fovea capitis femoris was aligned perpendicular to the antero-posterior (AP) axis on the axial views (right image). APM = anterior postero-medial, PPM = posterior postero-medial, PPL = posterior postero-lateral, APL = anterior postero-lateral, PAL = posterior antero-lateral, AAL = anterior antero-lateral, AAM = anterio antero-medial, PAM = posterior antero-medial. (Bottom): 3-dimensional renderings illustrating the 5 macro regions over which the morphometric parameters were statistically evaluated.

To create the volumes of interest for morphological evaluation within the trabecular bone, the spherical coordinate system was created at the centroid of the trabecular region (x_c_, y_c_, z_c_). Three hemispheres were created above z_c_, and along the SI axis, each with a depth of one third of the r_sphere_, representing the subchondral trabecular bone (STB), middle trabecular bone (MTB) and central trabecular bone (CTB) (Fig. 2). The STB and MTB hemi-annuli were further divided into superior and inferior regions, by an elevation angle (φ) of π/4 (Fig. 2), resulting in 5 macro regions: CTB, inferior (inf) and superior (sup) MTB and the inferior (inf) and superior (sup) STB (Fig. 2), which were analysed to evaluate primary morphometric variations. To further elucidate regional morphometric variations; in the axial view, the MTB and STB regions were divided into an additional eight “sub-regions” based on an azimuth angle (φ) = π/4 radians (Fig. 2). The regions were named according to their anatomic locations as follows: anterior postero-medial (APM), posterior postero-medial (PPM), posterior postero-lateral (PPL), anterior postero-lateral (APL), posterior antero-lateral (PLA), anterior antero-lateral (AAL), anterior antero-medial (AAM), posterior antero-medial (PAM); resulting in a total 37 VOIs per specimen (Fig. 2).

Once the VOIs were created, the following bone microarchitectural parameters were measured in the trabecular bone regions: bone volume fraction (BV/TV, %), trabecular thickness (Tb.Th, μm), trabecular separation (Tb.Sp, μm), trabecular number (Tb.N, mm^−1^), connectivity density (Conn.D, mm^−3^), and degree of anisotropy (DA). BV/TV was defined as the proportion of the VOI occupied by binarized bone tissue voxels; Tb.Th and Tb.Sp were computed without model assumptions, using the local sphere-fitting method^36^; Tb.N was calculated as fractional volume divided by the thickness. Conn.D is a parameter of trabecular connectivity normalised by the total volume. The index was developed to characterise the redundancy of trabecular connections, and is derived from the Euler number^38^, where a higher value indicates greater connectivity^39^. DA is a measure of how highly oriented substructures are within a given volume and was computed for the trabecular region within the entire femoral head only.

Bone cysts were identified by visual interrogation of VOIs that exhibited a standard deviation of Tb.Sp greater than 585 μm^40^. Osteophytes were characterised by identification of bone growths outside of the cortical shell.

Due to the small sample size, only trends will be presented in the manuscript, as example of the results that can be obtained from the developed approach.

## Results

**Figure 3:**
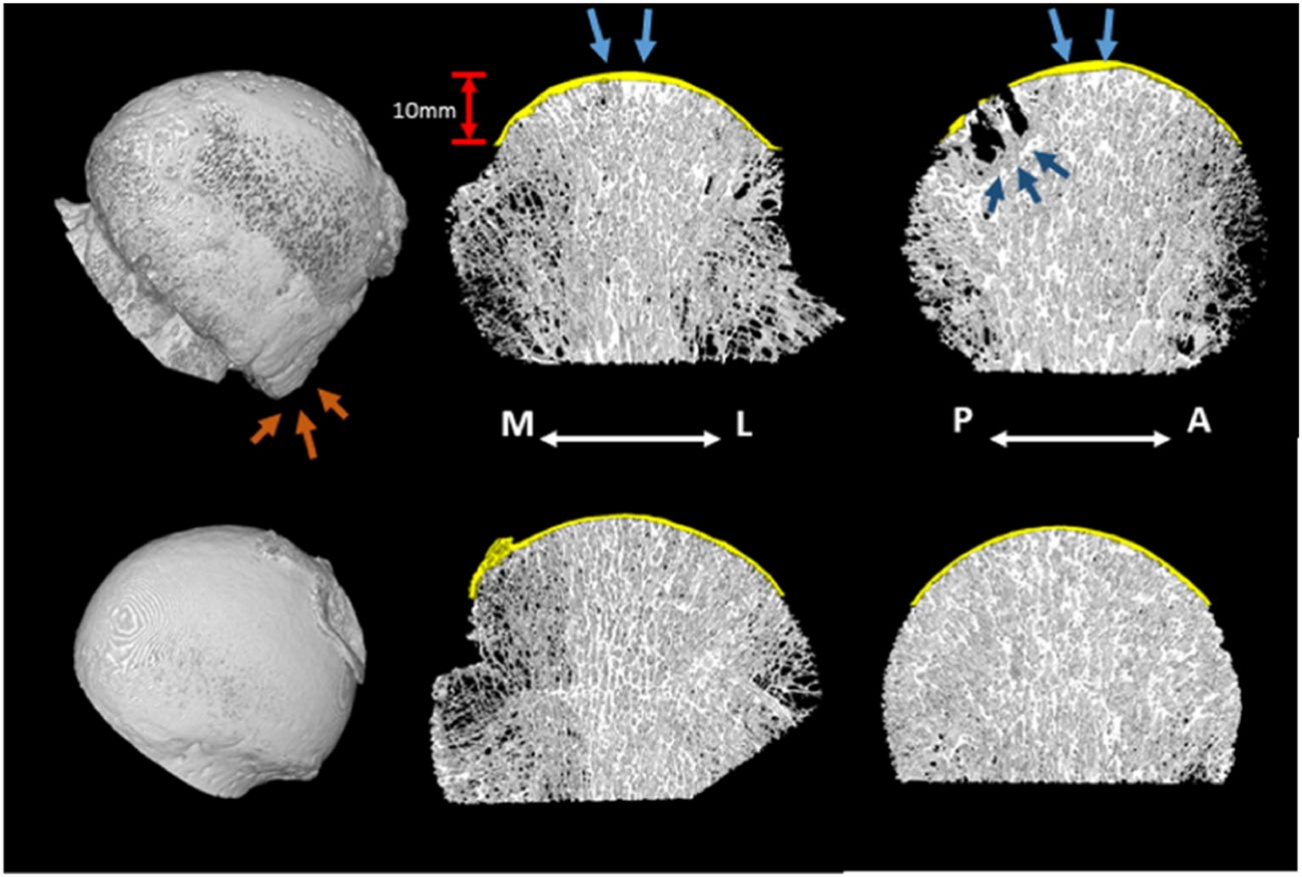
3D renderings of one of the OA (top) and HC (bottom) specimens. 2 mm slices in the coronal plane (centre) and sagittal plane (right) illustrate the presence of cysts and sclerotic bone (dark blue arrows) in the OA specimens. The SBP is shown in yellow and a thickening of the plate directly above the MTD (blue arrows) is evident in the OA specimens. A rendering of the entire femoral head (left) illustrates the significant osteophytosis in the OA specimens (orange arrows).

3D renderings of OA and HC specimen are shown in Fig. 3 to demonstrate the differences in morphology between the two groups.

### Osteophytes and bone cysts

All five OA specimens and none of the HC specimens presented with osteophytosis. The 3D rendering images in Fig. 3 illustrate typical osteophytosis in an OA specimen. The osteophytes were mostly located inferior to the centrum (i.e. below the level at which the morphometric analysis was performed). Osteophytes that were present above the centrum were located in the postero-lateral segments. Only one osteophyte extended into the superior region for one specimen, which was in the APM segment.

Subchondral bone cysts were found in four of the OA specimens and none in the HC specimens. The cysts were located posteriorly (APL, PPL and PPM) (Fig. 3), with only one specimen having cysts additionally in the APM, PAL and AAL regions, all of which were smaller compared to the other cysts within the specimen. The same specimen had subchondral bone cysts that extended into the middle trabecular bone region, all other bone cysts were confined to the subchondral trabecular bone layer. No specimen had cysts in the AAM and PAM regions.

### Cortical bone

**Figure 4:**
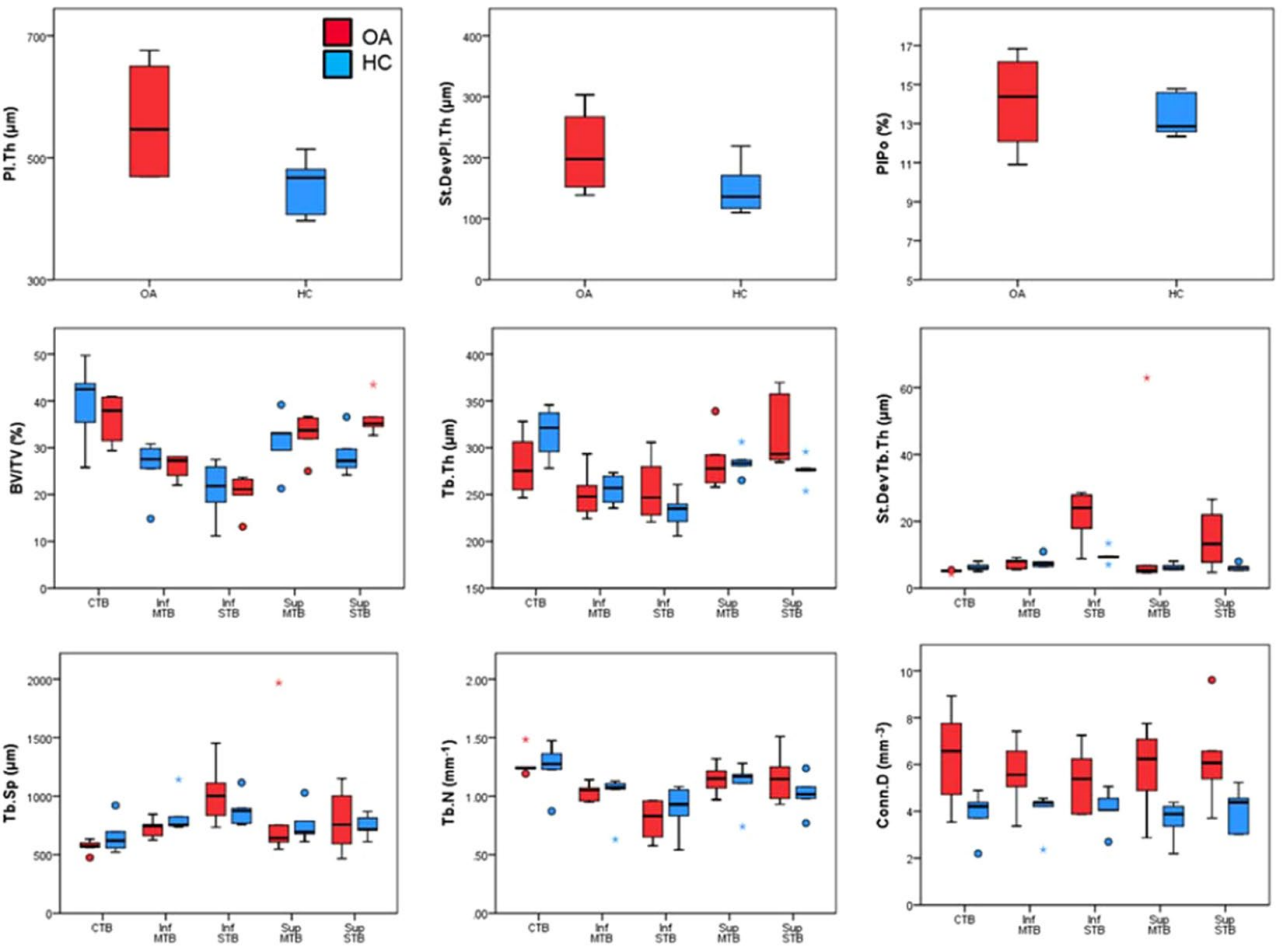
Boxplots of micro-structural properties measured within the subchondral bone plate (SBP) and the subchondral trabecular bone (STB) for OA (red) and HC (blue) specimens. The femoral head was divided into three hemispherical layers representing the central trabecular bone (CTB), middle trabecular bone (MTB) and subchondral trabecular bone (STB). The MTB and STB regions were divided into superior and inferior regions by elevation angle of π/4. (Circles denote outliers of 1.5 × IQR, star denotes extreme outlier >3.0 × IQR). A trend towards increased Pl.Th was observed in the OA specimens, along with increased variance in Pl.Th and increased Pl.Po. Trends of increased BV/TV, Tb.Th and Conn.D were observed in the OA specimens, but considering the low sample size these results obtained with the new analytical approach should be confirmed by analysing a larger number of specimens.

**Figure 5:**
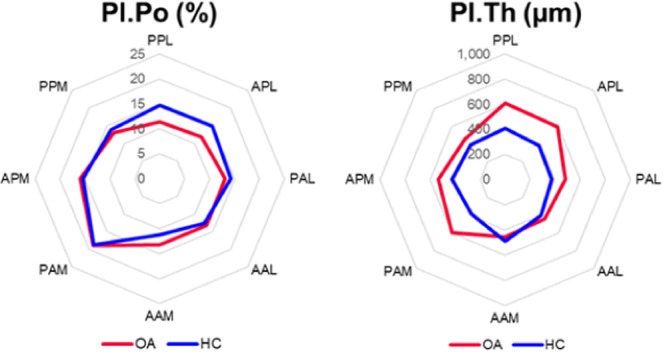
Regional distribution of structural properties in the subchondral bone plate (SBP). Increased plate thickness (Pl.Th) is evident in the medio-lateral plane for the OA specimens. No difference in plate porosity (Pl.Po) was observed between the two groups. (PPL = posterior poster-lateral; APL = anterior postero-lateral; PAL = posterior antero-lateral, AAL = anterior antero-lateral; AAM = anterior antero-medial; PAM = posterior antero-medial; APM = anterior postero-medial; PPM = posterior postero-medial).

**Figure 6:**
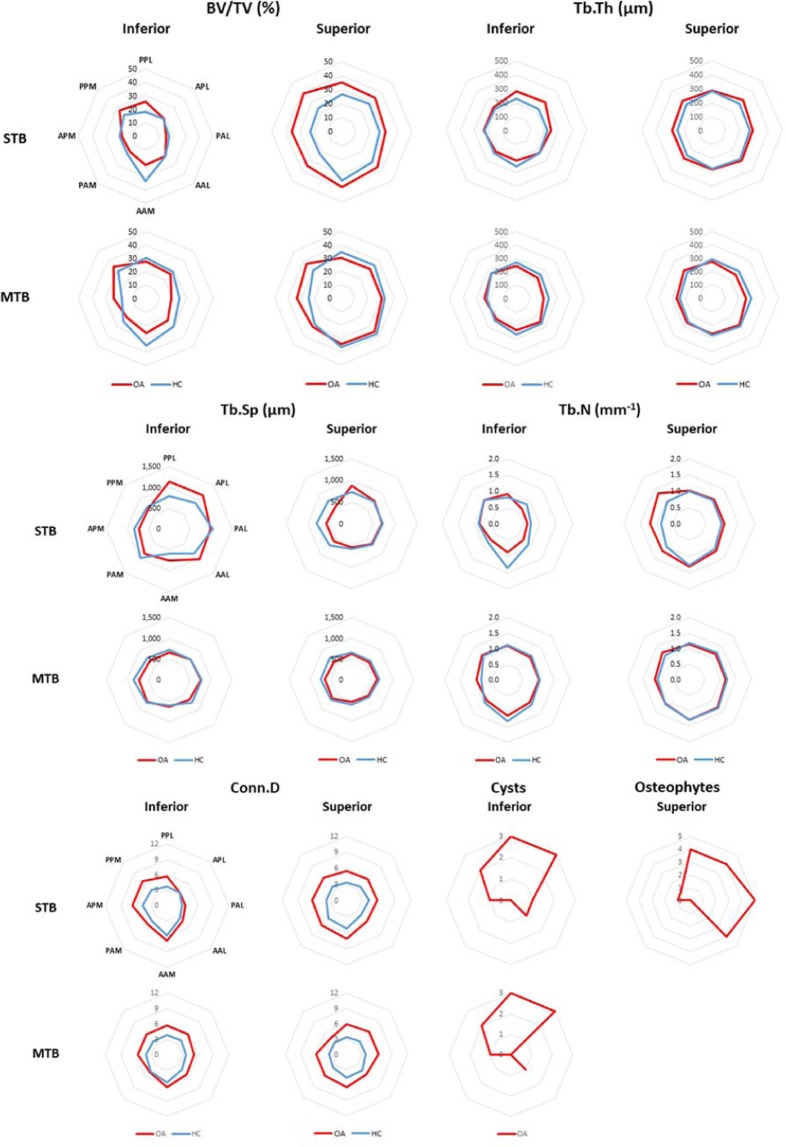
Regional distribution of the median value for the morphometric parameters evaluated within the trabecular bone for OA (red) and HC (blue) specimens. For each parameter, the measurements in the STB hemisphere are shown at the top, and the measurements in the MTB hemisphere are shown on the bottom; the inferior regions are shown in the left column, and the superior regions in the right column. The same regional classification was used for each graph but was reported only on the top left graph (PPL = posterior poster-lateral; APL = anterior postero-lateral; PAL = posterior antero-lateral, AAL = anterior antero-lateral; AAM = anterior antero-medial; PAM = posterior antero-medial; APM = anterior postero-medial; PPM = posterior postero-medial).

For one specimen of the OA group (82 years old male), it was not possible to delineate between the cortical and trabecular bone in the superior slices due to the absence of a clear difference in the morphology of the two microstructures; consequently, no cortical region was created for this specimen and the entire bone volume was included in the trabecular evaluation for the top 240 slices (i.e. 9.36 mm). Thus, the evaluation of cortical VOIs consisted of 5 HC specimens and 4 OA specimens. Figure 4 shows similar Pl.Po between the two groups and trends towards increased Pl.Th, increased variance in Pl.Th and increased variance in Pl.Po for the OA group. The median regional distribution of Pl.Po and Pl.Th is shown in Fig. 5. A greater variability in Pl.Th was observed among the 8 regions for the OA group (details in the individual plots for each specimen in the Supplementary Material). The increase in Pl.Th tended to occur in the medial and lateral segments (Fig. 6).

### Trabecular bone

Across all specimens, the process of alignment resulted in a median (min-max) deviation from the z-axis of the principle eigenvector of 5.2 degrees (1.0–22.0). The median deviation of the second and third eigenvectors from the AP and ML axes was 3.0 degrees (0.9–22.1) and 2.5 (0.4–19.0) degrees, respectively. The median DA computed for the full trabecular bone region was 0.44 (0.15–0.61) for the OA group, and 0.49 (0.23–0.62) for the HC group.

Trabecular morphometric parameters in the five macro-regions are shown in Fig. 4. In the subchondral bone region trends towards lower BV/TV, Tb.Th and Tb.N and higher Tb.Sp were observed compared to the central region. Our regional analyses on this limited sample size showed trends towards lower BV/TV, Tb.Th and Tb.N and increased Tb.Sp in the lower regions of the femoral head compared to the central and the superior regions. Higher variability in Tb.Th was observed for the OA group in the superior and inferior subchondral trabecular bone regions. All the parameters in the other sub-regions did not show any particular trend between the two groups.

The regional distribution of morphometric parameters is shown in Fig. 6 (regional distribution of individual specimens is available in the Supplementary Material). Consistent with evaluations over the global VOIs, a trend towards higher BV/TV for the OA was showed only in the superior subchondral trabecular bone regions, with peak of differences in the postero-medial (PPM) segment, in line with a trend towards higher Tb.N in the same region. On average, BV/TV was 43.4% lower (between 32.7% and 59.7%) in the inferior STB region compared to the superior STB region, whereas for the HC group BV/TV was 24.9% lower (between 12.8% and 53.8%) in the inferior STB region compared to the superior STB region. Conn.D tended to be higher for the OA group, especially in the central trabecular bone and inferior subchondral trabecular bone regions (Fig. 6).

## Discussion

The goal of this study was to develop an approach that would allow a comprehensive characterization of the micro-structural parameters in the cortical and trabecular bone of the human femoral head. The approach was applied to two groups of femoral heads from patient with severe osteoarthritis or healthy control to highlight the potential of the spatial analyses. Nevertheless, the reported results should be considered only as example of the potential of the approach and the observed trends might change with the increasing number of analysed specimens.

The potential of the methodology is highlighted by the identification of the morphometric properties in different sub-regions of the OA and HC specimens. It remains to be investigated if the results found in this study, like trends towards increased cortical Pl.Th, greater variance in Pl.Th, increased BV/TV, Tb.Th and ConnD in some specific regions of the subchondral bone of OA subjects in line with reports on late stage OA models^15,16,41^, are confirmed by the application of the approach to a larger number of specimens. A notable observation from this study was the higher variability in morphometric parameters for the OA group in the STB regions, specifically in terms of Tb.Th and Tb.Sp and Conn.D. The large standard deviation observed in Tb.Th measurements in the STB region suggests an increased heterogeneity in the bone architecture within this region and the thicker values are consistent with the sclerotic changes reported with OA^17–19,42^. These results are in line with the histopathology of OA, which is associated with higher bone density and more heterogeneous structural properties of the subchondral bone^43,44^. The subchondral trabecular bone region is subjected to the greatest impact under joint loading, and consequently higher BV/TV in the superior STB region compared to deeper bone has been reported^25,45^. Our results showed similar trends, with both OA and HC groups exhibiting higher BV/TV and Tb.Th in the superior STB regions compared to the superior MTB regions.

In the same region, the large variance in Tb.Sp is likely due to the presence of cysts (large Tb.Sp), which tended to be surrounded by sclerotic bone (small Tb.Sp). The outlier for Tb.Sp in the superior MTB region for one specimen was co-located with a large cyst, which comprised most of that VOI. In fact, the method implemented to identify cysts based on the standard deviation in Tb.Sp^40^ seems a valuable approach. Based on this criterion, all cysts were identified. In addition, six (out of 28) specimens were falsely classified as having cysts where they instead displayed a very low BV/TV. These low BV/TV regions were all located in the inferior subchondral trabecular bone region. In knee OA patients, Chiba, *et al*.^42^ noted that sclerotic changes in the medial regions were accompanied with osteoporotic changes in the lateral region. The authors attributed this to a shift in loading towards the medial joint, combined with unloading of the lateral aspect. It is possible that a similar shift in loading occurs in the femoral head; with the sclerotic changes that were observed in the superior STB region, the load may be directed more along the MTD, with a potential unloading of the inferior bone on the periphery.

Subchondral bone cists were present in all but one of the OA specimens. These lesions were associated with increased variability in Tb.Th; the only OA specimen that did not exhibit any cysts was the same OA specimen that had the smallest standard deviation in Tb.Th. The cysts were found in both the STD and MTD layers within the postero-lateral quadrant.

Osteophytes are osteo-cartilagenous tissues that form at the margins of OA joints. The precise function in the pathogenesis of OA is still unclear, it is not known whether they occur in response to altered joint mechanics, or as a side effect of the anabolic response to altered biomechanics that promotes chondrogenesis. The specimens from this study presented with mostly marginal osteophytosis, located laterally. One specimen exhibited epiarticular growths within the APM region, which is consistent with the most common location for epiarticular growths^46^.

This study had some limitations. The sample size was small for this study; however, this was a preliminary study to test a new approach to evaluating regional variations in local micro-architecture. Similar trends in bone changes were observed to what has been reported in the literature for late stage OA. Further studies with a larger sample size would validate the results and may help to identify region-specific microstructural changes, which could not be achieved with the current number of samples. The difference in age range for the two groups makes the comparison of the morphometric parameters between OA and healthy bones more complicated. Nevertheless, the large variability in most parameters for the OA group is consistent with the degree of degeneration of the joint, which seems not to affect only a specific region of the femoral head but to be more spread in the subchondral region. Furthermore, the evaluation of the cortical parameters depends on the ability of segmenting between cortical and trabecular bone in a reproducible way. This may not be possible in some of the cases due to the presence of large osteophytes or of other forms of tissue degeneration with high porosity of the cortical shell. Therefore, in order to provide detailed and generalizable characterization of the cortical bone tissue a large number of specimens with different degree of OA should be analysed. In addition, the anatomically named regions are somewhat arbitrary; the femoral heads were aligned according to the mean trabecular direction; and the fovea capitis, which may vary slightly across specimens. The subsequent regions are therefore named with respect to the loading orientation defined, which may not be consistent with the true anatomical alignment. Once these two issues are solved, our regional approach can be used to look for association between the regional microstructural properties of bone and external loading, both affected by OA. Finally, due to the storage method of the OA specimens, it was not possible to evaluate the degree of cartilage damage and how the damaged region correlated with the alignment of the specimen.

The methods developed herein provide a comprehensive and systematic investigation of microstructural differences within the femoral head between osteoarthritic and healthy control specimens. The results highlight the increased heterogeneity in the bone microarchitecture with osteoarthritis and suggest that these differences may be regionally specific. The subchondral bone plate appears thicker directly under the joint surface, with greater heterogeneity in osteoarthritic specimens. Likewise, the subchondral trabecular bone appears more connected with denser and thicker trabeculae closer to the joint surface, possibly accompanied by bone resorption in the inferior regions away from the principle loading axis. These results warrant further research to explore the localisation of microstructural changes and how these vary in different stages of osteoarthritis. Future work should also look to understand how STB changes and increased heterogeneity influences strain distributions under various loading conditions by using finite element method and/or digital volume correlation^47^. A similar approach can be used to characterize the effect of other pathologies such as osteoporosis, necrosis, bone metastases, etc. on the micro-architecture of the human femoral heads.

## Supplementary information

Supplementary information

## Data Availability

The datasets generated during and/or analysed during the current study are available from the corresponding author on reasonable request.
