# Supplementary material for: A new approach to comprehensively evaluate the morphological properties of the human femoral head: example of application to osteoarthritic joint
Source: Sci Rep. 2020 Mar 26;10:5538. doi: 10.1038/s41598-020-62614-7 (PMC7098981; doi:10.1038/s41598-020-62614-7)
Supplement: Supplementary file 1 — Supplementary information [file 41598_2020_62614_MOESM1_ESM.pdf]

A new approach to comprehensively evaluate the morphological properties of the human femoral head: example of application to osteoarthritic joint.

M. Ryan [a,b], L. Barnett [b], J. Rochester [c], J.M. Wilkinson [a], E. Dall'Ara [a,b] *

[a] Department of Oncology and Metabolism, Mellanby Centre for bone Research, University of Sheffield, UK

[b] INSIGNEO Institute for in silico Medicine, University of Sheffield, UK

[c] Academic Unit of Medical Education, Medical School, University of Sheffield, UK

Supplementary material

Tables

Table S1: Morphometric parameters in the trabecular VOIs of the superior (STB), middle (MTB) and central (CTB) hemispheres for OA and HC specimens (medians and ranges reported below).

Group	ROI	φ	BV/TV (%)	Tb.Th (µm)	Tb.Sp (µm)	Tb.N (µm)	Conn.D (mm^{-3})
**OA**	**STB**	1	21.13 (13.14-23.65)	247 (221-306)	1,001 (733-1,452)	0.83 (0.58-0.96)	5.39 (3.88-7.25)
	**STB**	2	35.18 (32.64-43.47)*	293 (285-370)	757 (466-1,1510	1.15 (0.93-1.51)*	6.07 (3.71-9.61)
	**MTB**	1	27.26 (21.98-28.14)	248 (224-293)	745 (625-845)	1.05 (0.95-1.14)	5.56 (3.37-7.42)
	**MTB**	2	33.69 (25.01-36.67)	277 (258-339)	641 (546-1,967)	1.15 (0.97-1.32)	6.24 (2.87-7.75)
	**CTB**		37.91 (29.38–41.01)	275 (247–328)	573 (476-633)	1.24 (1.19-1.48)	6.57 (3.54-8.93)
**HC**	**STB**	1	21.82 (11.17-27.49)	235 (206-261)	873 (758-1,115)	0.83 (0.04-1.05)	4.05 (2.70-5.06)
	**STB**	2	27.23 (24.18-36.58)	276 (254-296)	715 (611-868)	1.02 (0.77-1.24)	4.38 (3.03-5.23)
	**MTB**	1	27.56 (14.86-30.83)	257 (235-273)	755 (738-1,142)	1.07 (0.63-1.13)	4.38 (2.36-4.55)
	**MTB**	2	33.12 (39.19-32.71)	283 (265-306)	694 (622-1,029)	1.17 (0.74-1.28)	3.88 (2.19-4.38)
	**CTB**		42.51 (25.80-49.72)	321 (278-345)	619 (521-921)	1.27 (0.87-1.48)	4.21 (2.19-4.89)

φ = elevation angle, * $p < 0.05$

Figures

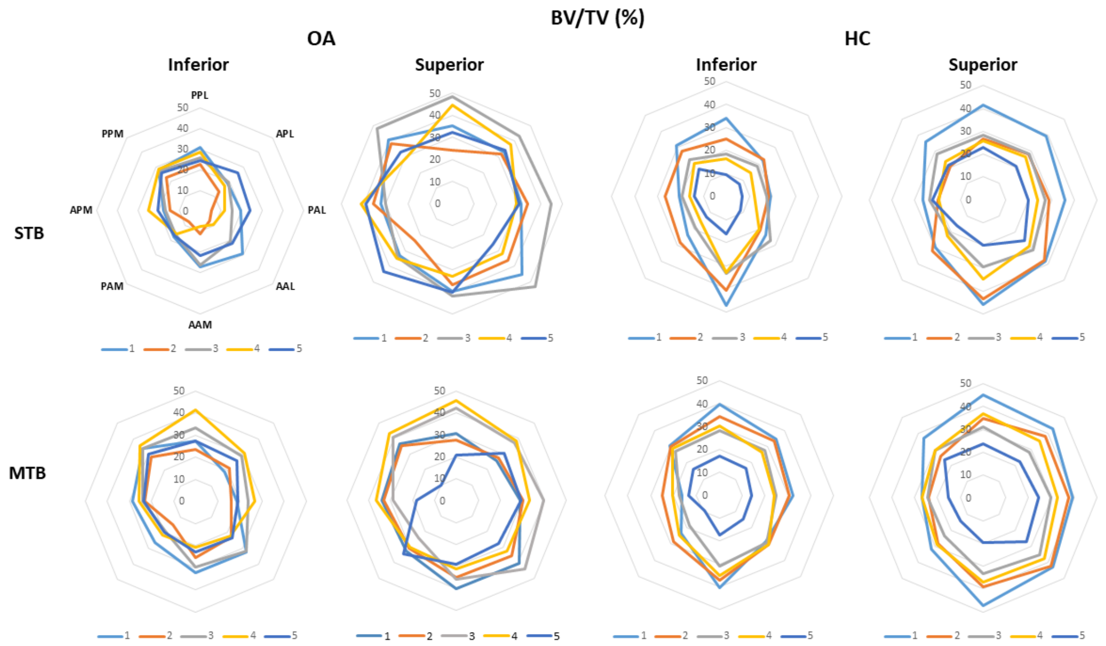

Figure S1: Regional distribution of the bone volume fraction (BV/TV) evaluated within the trabecular bone for each specimens. The measurements in the STB hemisphere are shown at the top, and the measurements in the MTB hemisphere are shown on the bottom; For each group (OA and HC) the inferior regions are shown in the left column, and the superior regions in the right column. The same regional classification was used for each graph but was reported only on the top left graph (PPL = posterior poster-lateral; APL = anterior postero-lateral; PAL = posterior antero-lateral, AAL = anterior antero-lateral; AAM = anterior antero-medial; PAM = posterior antero-medial; APM = anterior postero-medial; PPM = posterior postero-medial)

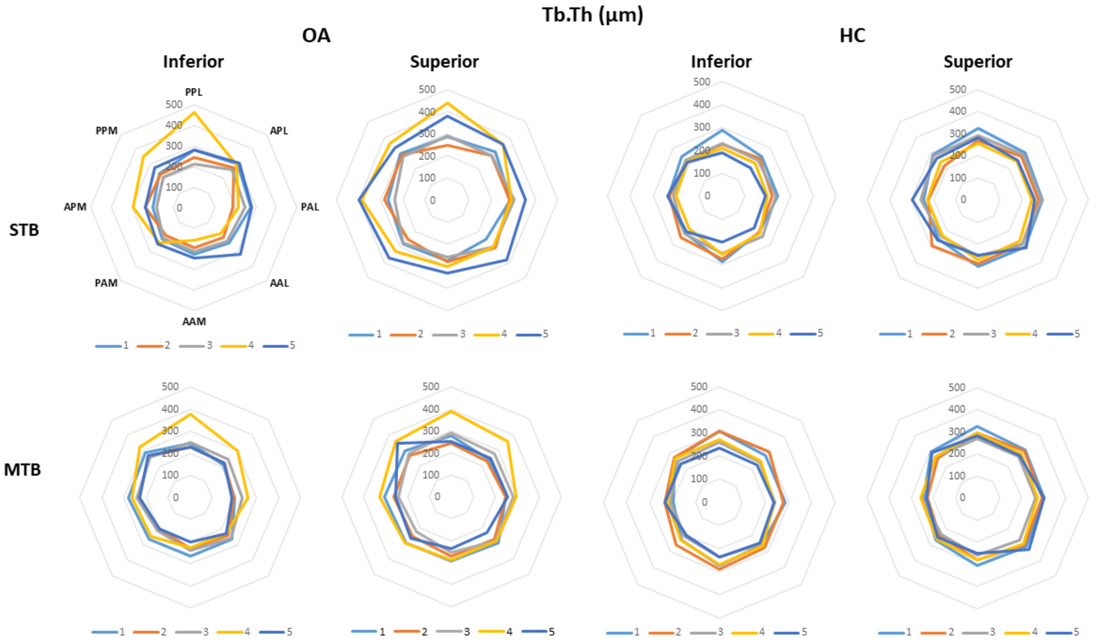

Figure S2: Regional distribution of trabecular thickness (Tb.Th) evaluated within the trabecular bone for each specimens. The measurements in the STB hemisphere are shown at the top, and the measurements in the MTB hemisphere are shown on the bottom; For each group (OA and HC) the inferior regions are shown in the left column, and the superior regions in the right column. The same regional classification was used for each graph but was reported only on the top left graph (PPL = posterior poster-lateral; APL = anterior postero-lateral; PAL = posterior antero-lateral, AAL = anterior antero-lateral; AAM = anterior antero-medial; PAM = posterior antero-medial; APM = anterior postero-medial; PPM = posterior postero-medial)

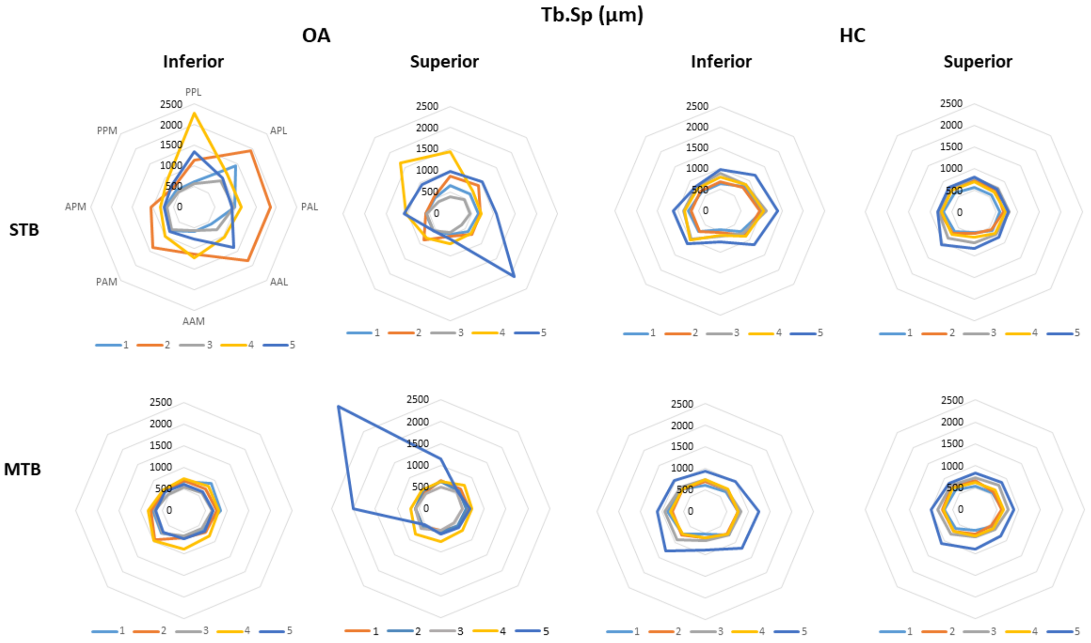

Figure S3: Regional distribution of trabecular spacing (Tb.Sp) evaluated within the trabecular bone for each specimens. The measurements in the STB hemisphere are shown at the top, and the measurements in the MTB hemisphere are shown on the bottom; For each group (OA and HC) the inferior regions are shown in the left column, and the superior regions in the right column. The same regional classification was used for each graph but was reported only on the top left graph (PPL = posterior poster-lateral; APL = anterior postero-lateral; PAL = posterior antero-lateral, AAL = anterior antero-lateral; AAM = anterior antero-medial; PAM = posterior antero-medial; APM = anterior postero-medial; PPM = posterior postero-medial)

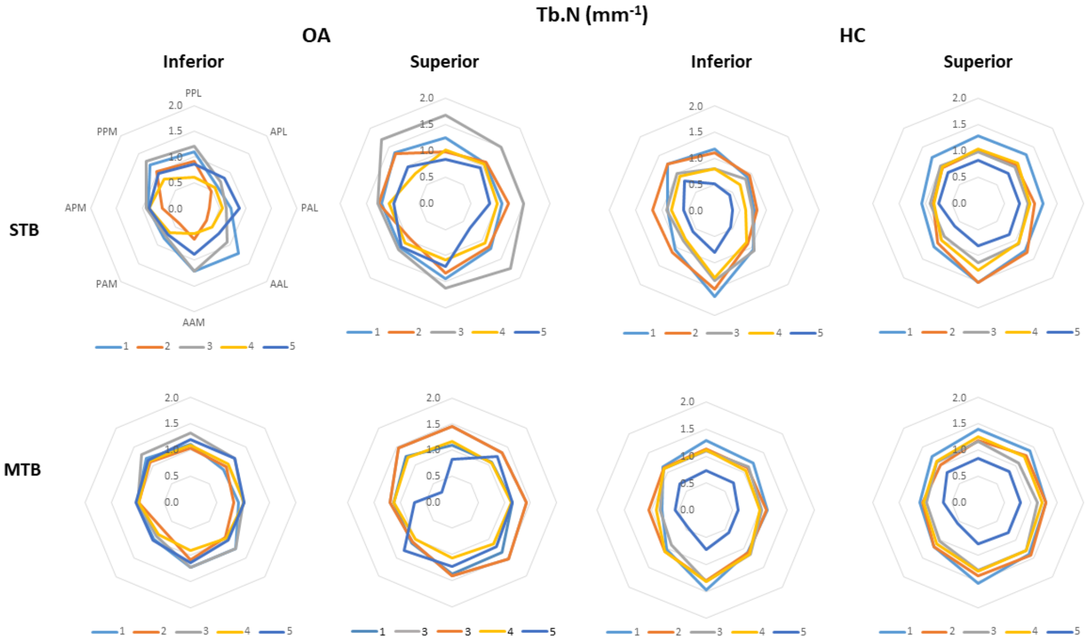

Figure S4: Regional distribution of trabecular number (Tb.N) evaluated within the trabecular bone for each specimens. The measurements in the STB hemisphere are shown at the top, and the measurements in the MTB hemisphere are shown on the bottom; For each group (OA and HC) the inferior regions are shown in the left column, and the superior regions in the right column. The same regional classification was used for each graph but was reported only on the top left graph (PPL = posterior poster-lateral; APL = anterior postero-lateral; PAL = posterior antero-lateral, AAL = anterior antero-lateral; AAM = anterior antero-medial; PAM = posterior antero-medial; APM = anterior postero-medial; PPM = posterior postero-medial)

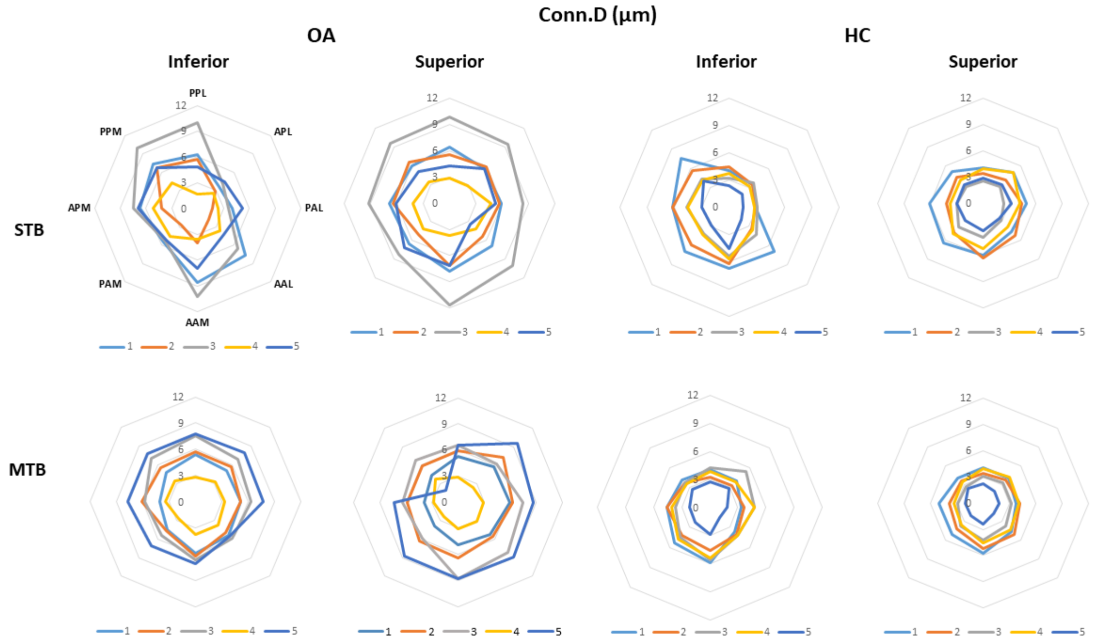

Figure S5: Regional distribution of trabecular connectivity density (Conn.D) evaluated within the trabecular bone for each specimen. The measurements in the STB hemisphere are shown at the top, and the measurements in the MTB hemisphere are shown on the bottom; For each group (OA and HC) the inferior regions are shown in the left column, and the superior regions in the right column. The same regional classification was used for each graph but was reported only on the top left graph (PPL = posterior poster-lateral; APL = anterior postero-lateral; PAL = posterior antero-lateral, AAL = anterior antero-lateral; AAM = anterior antero-medial; PAM = posterior antero-medial; APM = anterior postero-medial; PPM = posterior postero-medial)

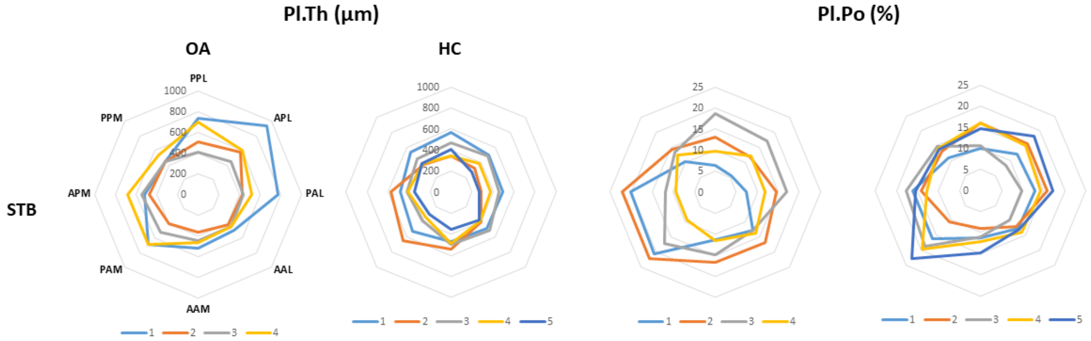

Figure S6: Regional distribution of plate thickness (Pl.Th) and plate porosity (Pl.Po) evaluated within the subchondral plate. The measurements in the STB hemisphere are shown at the top, and the measurements in the MTB hemisphere are shown on the bottom; For each group (OA and HC) the inferior regions are shown in the left column, and the superior regions in the right column. The same regional classification was used for each graph but was reported only on the top left graph (PPL = posterior poster-lateral; APL = anterior postero-lateral; PAL = posterior antero-lateral, AAL = anterior antero-lateral; AAM = anterior antero-medial; PAM = posterior antero-medial; APM = anterior postero-medial; PPM = posterior postero-medial)
